# Association of Timely Goals of Care Discussions with Hospitalizations Due to Infections in Nursing Homes

**DOI:** 10.1177/26892820251410099

**Published:** 2026-03-19

**Authors:** Jung A. Kang, Andrew W. Dick, Laurent G. Glance, Lara Dhingra, Patricia W. Stone

**Affiliations:** 1 Columbia University School of Nursing, New York, New York, USA.; 2 Health Unit, RAND Corporation, Boston, Massachusetts, USA.; 3 Departments of Anesthesiology and Perioperative Medicine; Public Health Sciences, University of Rochester School of Medicine, Rochester, New York, USA.; 4 MJHS Institute for Innovation in Palliative Care, New York, New York, USA.; 5 Albert Einstein College of Medicine, Bronx, New York, USA.

**Keywords:** end-of-Life care, infection-related hospitalizations, nursing home, timely goals of care (TGOC)

## Abstract

**Background:**

Hospitalizations from infections are common among nursing home (NH) residents and can lead to burdensome treatments that may not align with residents’ preferences for care. Although goals of care discussions can ensure that residents’ wishes are honored, little is known about the impact of timely discussions on residents’ infection outcomes.

**Objectives:**

To determine the association between timely goals of care (TGOC) discussions and infection-related hospitalizations among NH residents.

**Design:**

Retrospective cross-sectional analysis.

**Setting/Subjects:**

The sample consisted of 892 NHs who participated in a 2018 national survey in the United States that included a TGOC index.

**Measurements:**

A facility-level TGOC index (scored 0–18) was categorized into two levels (0–5, 6–18). Hospitalizations from infections were identified using CMS claims data. Multivariable logistic regression was used to examine the association between infection-related hospitalization and TGOC intensity.

**Results:**

A total of 867 NHs with 988,502 resident observations were analyzed. The mean TGOC index was 13.6 (SD = 5.1); about 10% fell in the lower TGOC category (0–5). Residents were, on average, 82 (SD = 9) years old; mostly female (63.9%), and White (84.6%). In fully adjusted models, residents in facilities with higher TGOC engagement (scores 6–18) had lower odds of infection-related hospitalization compared to those in facilities with lower engagement (scores 0–5) (AOR 0.84, 95% CI: 0.72–0.99, *p* < 0.05). Sensitivity analyses using the original four-level TGOC index produced consistent results.

**Conclusions:**

TGOC discussions are associated with reduced infection-related hospitalizations in NHs. Future research should examine how facility policies and staff training shape TGOC implementation.

## Key Message

This national study examined the association between timely goals of care (TGOC) discussions and infection-related hospitalizations in nursing home residents. Greater TGOC engagement was associated with reduced infection-related hospitalization rates. Strategies to implement TGOC discussions may reduce unnecessary hospital transfers at the end of life.

## Introduction

Nursing home (NH) residents experience a high risk of infections near the end-of-life (EOL),^1,2^ highlighting the importance of medical decision-making to manage care and optimize resident outcomes. As function declines at the EOL, the immune response may become compromised, increasing residents’ susceptibility to infections.^3–6^ Before the COVID-19 pandemic, NHs experienced up to 2.7 million infections annually, leading to over 380,000 residents deaths each year.^1,2^

Infections are a leading precipitant of NH transfers near the EOL and often represent avoidable, burdensome care.^7,8^ About half of transfers for suspected infection are potentially avoidable.^9^ Infections account for a large share of NH hospitalizations and an even higher proportion among residents with advanced dementia, congestive heart failure (CHF), or chronic obstructive pulmonary disease (COPD).^10,11^ These conditions heighten vulnerability: dementia increases aspiration risk and impedes symptom reporting; CHF and COPD limit cardiopulmonary reserve, making even minor infections destabilizing.^11,12^ Unnecessary infection-related transfers accelerate functional and cognitive decline, increase costs, and often conflict with residents’ stated wishes.^11–13^ These realities make infection management an actionable focal point for quality improvement in EOL care.

An important factor in infection-related hospitalization at the EOL is how effectively NHs elicit and honor the resident’s and family’s values and preferences for health care. These communications involve conversations between health care providers, residents, and their families about current or future health care decisions, including infection management toward the EOL. Timely goals of care (TGOC) discussions provide residents and families with sufficient time to understand the resident’s condition, prognosis, and treatment options, which promotes shared decision-making.^14–19^ In this study, TGOC denotes proactive, infection-focused conversations about values and treatment preferences that occur early and are revisited at clinical triggers, rather than only at the moment of transfer. We measured TGOC discussions with a validated 4-item index adapted from the Palliative Care Survey,^20^ capturing how early and often facilities hold TGOC discussions at key junctures (on admission, during care-plan meetings, with condition changes, and after acute events) focusing on infection-related preferences (e.g., antibiotic use).

However, these discussions are often delayed or infrequent, limiting opportunities for informed, shared decision-making and increasing the risk of burdensome, unwanted hospital transfers.^10^ Despite its importance, there is limited knowledge about how TGOC discussions, particularly related to infection management, impact EOL outcomes in NHs.^21^ Therefore, this study aims to determine the association between TGOC discussions and hospitalizations due to infections in NH residents. We hypothesized that residents living in NHs with more frequent and earlier discussions will have fewer hospitalizations from infections compared to those living in NHs with less frequent and delayed TGOC discussions.

## Methods

This study was a retrospective cross-sectional, secondary analysis of data acquired from a national palliative care survey of United States NHs. The Columbia University Irving Medical Center Institutional Review Board approved the study (IRB-AAAU3141).

### Data sources

A 2018 national survey of NHs and five national datasets were linked including: the 2018 Minimum Data Set 3.0 (MDS), the Provider of Service (POS) file, the American Community Survey (ACS), the 2018 Medicare Provider and Analysis Review (MedPAR), and NH payroll data. For the survey, NHs were randomly selected from the Certification and Survey Provider Enhanced Reports data, stratified by state, and urban or rural status.^10^ To ensure the sample was nationally representative, the parent study excluded specialty NHs, those primarily providing sub-acute rehabilitation, hospital-affiliated facilities, and any with an annual census below 30 or above 900 residents. In 2018, 892 NH Director of Nurses completed and returned surveys, yielding a response rate of 49%. Population weights were applied, rendering the sample representative of the national NH population, which encompasses over 15,381 NHs across the United States.^10^

The MDS 3.0 data contain comprehensive information on resident-level characteristics from all NHs certified by the Centers for Medicare and Medicaid Services, which represents more than 95% of all NHs nationally.^22^ The MDS includes multiple resident-level characteristics, including demographics, functional status (e.g., Activities of Daily Living [ADL]), cognitive impairment level (e.g., Cognitive Function Scale [CFS]), and race and ethnicity. The POS file contains facility-level characteristics (bed size, ownership, provider type, and urban-rural location), while payroll data provide facility-level staffing information. The Social Deprivation Index (SDI), provided by the ACS, was aggregated at the Zip Code Tabulation Area level. The MedPAR data included inpatient admissions, while the NH final action stay records documents procedures, diagnoses (using International Classification of Diseases [ICD] 10-codes), diagnostic-related groups, and length of stay.

### Conceptual framework and variables

**FIG. 1.**
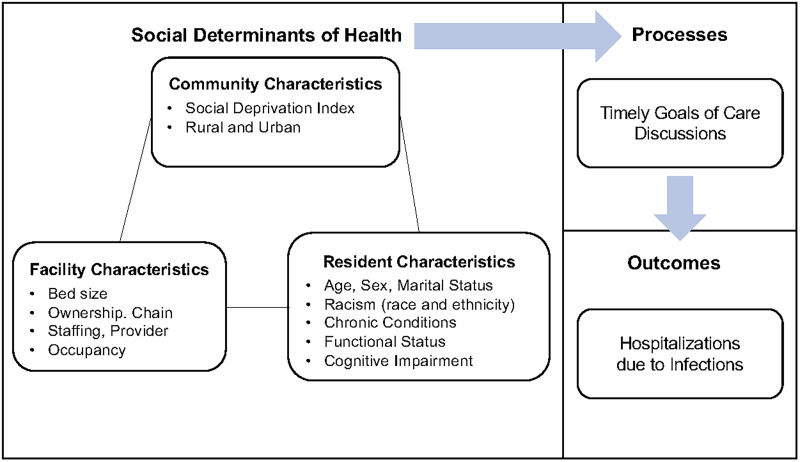
An Integrated Conceptual Model using the Donabedian Model and Minority Access to End-of-Life Care.

Our integrated conceptual framework (Fig. 1) combined the Donabedian Model’s^23^ focus on health care quality dimensions (structures, processes, and outcomes) with the Minority Access to EOL Care model.^24^ This framework served as the foundation for our study’s approach and variable selection. Central to our analysis is the examination of a wide range of resident-, facility-, and community-level characteristics.^25^

The key dependent variable in this secondary analysis was an indicator [yes/no] for hospitalizations due to infections in the last 90 days of life.^26,27^ Infections were identified based on ICD-10 codes in the MedPAR file. Hospitalizations were classified as due to infection if the infection was indicated as the MedPAR admitting diagnosis or as the primary diagnosis (the first of 25 diagnosis codes) and was present on admission. The list of infection-related ICD-10 codes and coding for hospitalizations due to infection has been developed by the research team, used in multiple papers and is available upon request.^11,12^

**FIG. 2.**
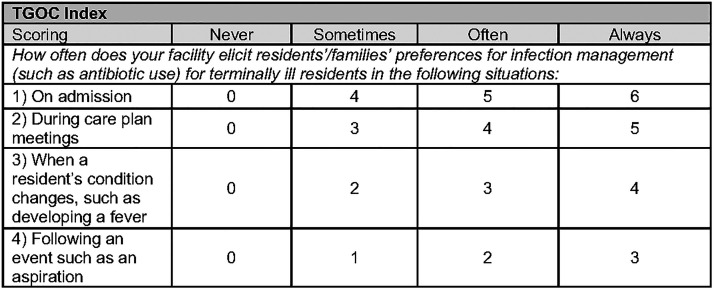
TGOC Index and Weighted Score. TGOC, timely goals of care.

The main independent variable in this study was the facility-level TGOC index, adapted from the Palliative Care Survey^20^ used in the parent study.^10,20^ We defined TGOC discussions as structured conversations with residents and/or families about values, infection management (e.g., antibiotic use), and potential hospital transfer that are (a) initiated early in the stay and (b) revisited when clinical status changes. TGOC was measured at the facility level using a four-item index. These items examine how often NHs engage residents and their families in discussions related to infection management across four key domains: (1) on NH admission, (2) during care plan meetings, (3) when a resident’s condition changes, and (4) following a medical event. Each item is scored on a 4-point Likert scale, with responses ranging from “Never” to “Always.” The four items were summed to calculate a facility-level index score, which ranges from 0 (minimum) to 18 (maximum) (see Fig. 2). Higher scores indicate more frequent and earlier engagement in TGOC discussions. The index items were previously validated in the original study^20^ and have shown good internal consistency (Cronbach’s alpha = 0.82). We used this scoring method as outlined in the prior study^28^ to maintain consistency across analyses. The use of this index allows for a clear and interpretable measure of the extent to which facilities engage in goals of care discussions with residents and families, which is critical for understanding the relationship between the timeliness of these discussions and hospitalization outcomes.

Resident characteristics from the MDS included race and ethnicity, age, sex, and marital status. Additionally, we adjusted for residents’ functional status (e.g., ADLs) and degree of cognitive impairment (e.g., CFS); the former quantifies functional independence, ranging from 0 (complete independence) to 16 (total dependence) and was categorized into quartiles. CFS ranges from 1 (intact) to 4 (severe impairment).

Facility characteristics from the POS data included bed size (<50, 50–100, >100), ownership (for-profit, not-for-profit, or government-owned), provider type (Medicare, Medicaid, or both), and chain status (part of a chain or independent). To assess the impact of nursing staff availability, we created measures of staffing hours per resident and occupancy rates derived from publicly available payroll data. The staffing measures included registered nurses, director of nurses (DONs), and licensed practical nurses hours per resident day and categorized by quartiles. A measure of occupancy rates (i.e., a percentage of occupied beds categorized by quartiles) was also included.

Community characteristics from ACS included the SDI (grouped by quartiles). The SDI incorporates key socioeconomic status (SES) indicators: income level, employment status, education level, housing conditions, family structure, vehicle availability, and language barriers. These indicators collectively provide a nuanced understanding of the social and economic factors influencing the effectiveness of TGOC discussions and their role in mitigating hospitalization rates.^29–31^ The SDI ranges from 0 to 100, with higher values indicating greater social deprivation. It is a well-validated metric of SES that predicts health care access and need.^32^ Both variables were included in the models to quantify the socioeconomic variation in health outcomes. Location (rural and urban) of the NH county was derived from POS.

### Study sample

The study sample included residents with an NH stay longer than 100 days (long-term), and older adults (>64 years of age) in NHs who participated in the 2018 survey.^33^

### Data analysis

After linking the data, descriptive statistics were examined to determine the distribution and frequencies of the variables. To facilitate interpretation while capturing meaningful variation in facility-level goals of care practices, we categorized the TGOC index into two levels: low TGOC (0–5) and high TGOC (6–18). This binary categorization was chosen based on observed distribution patterns and model performance in sensitivity analyses (Supplementary Appendix Table SA1, Figs. SA1, SA2). It simplifies interpretation and allows clearer comparisons across facilities with varying engagement in goals of care discussions. In a sensitivity analysis, we retained four TGOC index categories (0–5, 6–10, 11–15, and 15–18) to assess whether a more granular categorization would yield additional insights.

To understand the variation in characteristics of the overall sample and across TGOC index score categories, we conducted descriptive analyses of resident-, facility-, and community-level characteristics stratified by TGOC index categories. *P* values were calculated using Pearson’s chi-square or ANOVA, as appropriate, to identify differences across these categories. Next, multivariable logistic regression models were estimated to control for other covariates. To examine the hypothesis, multivariable logistic regression models at the resident level were estimated with standard errors clustered at the NH level to account for correlations within NHs. We used Stata version 17 to perform all analyses. Two-tailed tests with alpha = 0.05 were used.

## Results

In total, survey data from 892 NHs were merged with other datasets. Data from 25 NHs were excluded from the analysis due to discrepancies in matching data points across different datasets, resulting in a final sample of 867 NHs with a total of 988,502 NH resident observations for analysis. The mean TGOC index for the sample was 13.6 (SD = 5.1); the distribution showed that about 10% of observations fell into the lower category of TGOC index scores (Table 1, Supplementary Appendix Table SA1). In Table 1, the distributions for all variables varied across the levels of the TGOC index. Compared with all other facilities (TGOC ≥6), those with TGOC 0–5 were more often rural, served higher shares of Black and Hispanic residents, and were in more socioeconomically deprived communities (higher SDI). They were also modestly less likely to be part of a chain. Differences in ownership mix and bed size were small (see Table 1 for key contrasts and Supplementary Appendix Table SA2 for the full distribution). The average age of residents was 82.2 years (SD = 8.8), most were White (84.6%), female (63.9%), and widowed (46.1%). Approximately 45.9% of residents were categorized as having severe cognitive impairment (CFS score = 4) and moderate functional dependence, with ADL scores averaging 6.4 (SD = 4.7). More than half (53.3%) of the NHs in the sample had between 100 and 200 beds, were for-profit (62.4%), part of a chain (54.3%), located in urban areas (79.4%), and had residents who were both Medicare and Medicaid beneficiaries (97.4%). Additionally, the SDI for the communities where these NHs were located had an average score of 50.6 (SD = 27.0), indicating a mid-range level of social deprivation within the index’s possible range.

**Table 1.** Resident, Nursing Homes, and Community Characteristics in Sample. (867 NHs, 988,502 Observations)

Variables		TGOC index		*p*
	All	0–5	6–18	
TGOC Index (0–18) Mean (SD)	13.6 (5.1)	1.8 (2.0)	14.8 (3.5)	*p* < .001
*N* (%)	988,502 (100)	96,929 (9.8)	891,573 (90.2)	*p* < .001
Resident-level Characteristics				
Race and Ethnicity *n* (%)^a^				
White	836,152 (84.6)	79,254 (81.8)	756,898 (84.9)	
Black	83,608 (8.5)	11,470 (11.8)	72,138 (8.1)	
Asian	22,565 (2.3)	1,358 (1.4)	21,207 (2.4)	
American Indian Alaska Native	6,700 (0.7)	191 (0.2)	6,509 (0.7)	
Native Hawaiian Pacific Islander	4,012 (0.4)	516 (0.5)	3,496 (0.4)	
Hispanic	35,465 (3.6)	4,140 (4.3)	31,325 (3.5)	*p* < .001
Age mean (SD)	82.2 (8.8)	81.6 (8.7)	82.0 (8.8)	*p* < .001
Female *n* (%)^a^	631,946 (63.9)	59,290 (61.2)	572,656 (63.2)	*p* < .001
Facility-level Characteristics				
Bed size *n* (%)^a^				
<100	309,599 (31.3)	32,021 (33.0)	277,578 (31.1)	
100–200	526,403 (53.3)	53,139 (54.8)	473,264 (53.1)	
>200	152,500 (15.4)	11,769 (12.1)	140,731 (15.8)	*p* < .001
Ownership *n* (%)^a^				
For profit	616,356 (62.4)	57,688 (59.5)	558,668 (62.7)	
Not-for-profit	310,517 (31.4)	29,259 (30.2)	281,258 (31.6)	
Government	61,629 (6.2)	9,982 (10.3)	51,647 (5.8)	*p* < .001
Chain affiliated *n* (%)^a^	537,055 (54.3)	48,478 (50.0)	488,577 (54.8)	*p* < .001
Located in Rural *n* (%)^a^	203,888 (20.6)	25,376 (26.2)	178,512 (20.0)	*p* < .001
Provider *n* (%)^a^				
Medicare only	21,430 (2.2)	2,491 (2.6)	18,939 (2.1)	
Medicaid only	4,715 (0.5)	317 (0.3)	4,398 (0.5)	
Medicare and Medicaid	962,357 (97.4)	94,121 (97.1)	868,236 (97.4)	*p* < .001
Community-level Characteristics				
SDI Score (1–100) mean (SD)	50.6 (27.0)	55.9 (25.7)	50.0 (27.0)	*p* < .001

*p* Values were calculated using Pearson’s chi-square or ANOVA, as appropriate.

Full descriptive data by all four TGOC categories appear in Supplementary Appendix Table SA1.

a column percentage.

ADL, activities of daily living; CFS, cognitive function scale; SD, standard deviation; SDI, Social Deprivation Index; SES, socioeconomic status; TGOC, timely goals of care.

The results of the fully adjusted model are in Table 2. There was a significant association between TGOC and hospitalization due to infection (AOR = 0.84, 95%CI: 0.72–0.99, *p* < 0.05). This supports the hypothesis that higher TGOC engagement is associated with fewer hospitalizations due to infection. The fully adjusted model, including all resident, facility, staffing, and community covariates, is presented in Supplementary Appendix Table SA3.

**Table 2.** AOR For Hospitalization Due to Infection in Nursing Home Residents

Variables	Model		
	AOR	RSE	95% CI
TGOC Index (0–18)			
0–5	ref		
6–18	0.84*	0.07	0.72, 0.99

Adjusted for resident factors (age, sex, race/ethnicity, marital status, cognition, ADL dependence), facility factors (bed size, ownership, chain status, urban/rural location, payer mix, staffing hours for DONs/RNs/LPNs, occupancy), and community SDS (Social Deprivation Index).

*p < .05; **p < .01; ***p < .001.

The results of the sensitivity analysis with the four-category TGOC index (0–5[ref], 6–10, 11–15, and >15) and hospitalization due to infections are in Supplementary Appendix Table SA4. Compared to the lowest TGOC category (0–5), the 6–10 (AOR = 0.80, 95% CI: 0.66–0.97, *p* < .05) and >15 (AOR = 0.84, 95% CI: 0.71–0.99, *p* < 0.05) groups were significantly associated with lower odds of hospitalization, while the 11–15 group (AOR = 0.87, 95% CI: 0.73–1.05, *p* > 0.05) was not statistically significant. However, all three higher TGOC categories had substantively similar point estimates, and none were statistically significantly different from each other (Wald test, *p* = 0.11). Taken together, these findings indicate that once a facility surpasses the lowest TGOC threshold, any additional increase in engagement confers a comparable reduction in infection-related hospitalizations.

## Discussion

Using national data on 988,502 NH residents, we found that residents in NHs with greater implementation of TGOC discussions experienced 16% lower odds of infection-related hospitalizations after accounting for resident, facility, and community factors. We also found that the benefit of TGOC discussion leveled off at low levels of TGOC discussion, suggesting that even low levels of TGOC discussions are associated with a lower risk of infection-related hospitalizations.

Our results extend a growing evidence base linking advance care planning and goals of care discussions to better EOL outcomes.^34^ Large reviews and clinical trials have consistently shown that structured goals of care discussions improve quality of life, decrease unwanted aggressive treatments, and increase documentation of patient values in both acute-care and long-term-care settings.^34,35^ More recently, a national cross-sectional study found that integrating palliative care principles into infection management reduced infection-related transfers among residents with advanced illness, underscoring the specific relevance of goals of care to infectious-disease decisions at the EOL.^11^ Although our data predate the COVID-19 era, they provide an important baseline of facility practices immediately before substantial changes in NH operations and policy. Our findings are consistent with more recent work showing that facility-level processes and capacity shape transfer decisions and infection outcomes in the United States NHs (e.g., studies of COVID-era disparities and staffing/transfer patterns).^36–38^ Our work builds on these findings by demonstrating that even modest increases in TGOC engagement within routine NH operations can meaningfully decrease infection-related hospitalizations.

TGOC discussions may prevent potentially avoidable hospitalizations in several ways.^9^ First, explicit documentation of resident and family preferences enables bedside clinicians to manage infections within the NHs, especially when the burden of hospitalization outweighs the benefits, such as for frail residents who decline intravenous antimicrobials or mechanical ventilation.^9^ Evidence from treat-in-place programs, such as the Missouri lower respiratory tract infection pathways and the Dutch NH model shows that protocolized on-site management with after-hours support can safely reduce transfers and antibiotic use.^39^ Second, facilities committed to TGOC often invest in staff training, after-hours coverage, and protocols that collectively raise the threshold for transfer.^40,41^ Finally, avoiding preventable hospitalization for infections may prevent the downstream cognitive and functional decline that frequently follows acute transfers among older adults.^12,42–45^Together, our findings and prior studies suggest that wider adoption of structured TGOC programs could meaningfully reduce burdensome transfers. At a policy level, CMS quality measures and survey processes might incorporate TGOC engagement metrics alongside existing infection-control and hospitalization indicators. Moreover, TGOC programs are likely to function best in concert with high-quality infection-prevention infrastructure, antibiotic-stewardship initiatives, and culturally responsive communication training in serious illness discussions. Importantly, consistent with critiques of advance care planning, the impact of TGOC will depend not only on “having the conversation” but on its timing, specificity, and translation into actionable orders supported by systems that honor them (e.g., workflow prompts, after-hours protocols, and order sets).^46^ Our TGOC index captures timeliness and frequency but not conversation quality or implementation (e.g., time-stamped encounters); thus, TGOC may operate as a marker of broader facility culture and capability rather than the sole causal mechanism.

Further work should examine which TGOC elements, such as timing (e.g., on admission), frequency of review, trigger-based updates, or involvement of specific disciplines, drive the largest gains. Mixed-methods research could reveal how frontline staff operationalize “high TGOC” and identify barriers in facilities serving racially and ethnically minoritized populations. Pragmatic trials that integrate real-time clinical decision support with TGOC prompts could test causal effects on infections, hospice utilization, and quality of life scores. Finally, leveraging natural-language processing of progress notes may help capture undocumented goals of care nuances and monitor fidelity over time.

## Limitations

This study has several limitations. First, the secondary and cross-sectional analysis limits causal inference. Second, although the TGOC index is grounded in prior literature, it has not undergone formal psychometric or predictive validity testing, raising the possibility of measurement error or misclassification bias. Third, the index captures the presence of specific practices but cannot fully reflect the quality, depth, content, or consistency of discussions within and across facilities, nor differences in staff preparedness, skills, and training. Because the TGOC index measures timing/frequency rather than conversation content, it may not fully reflect whether antibiotic plans (e.g., route, duration) or explicit transfer directives were set; future instruments should capture these elements to better link TGOC content with hospitalization decisions. The use of 2018 data may not reflect current NH practices. While it remains the most recent national dataset on TGOC practices, care models and regulations may have changed since then. Future research should explore more recent data. TGOC scores are self-reported by DONs and may introduce potential social-desirability bias. Finally, the parent study formally compared survey responders with nonresponders using the Centers for Medicare & Medicaid Services Certification and Survey Provider Enhanced Reports national file, which enumerates all CMS-certified NHs and key structural characteristics, allowing us to assess potential selection bias.^10^

## Conclusion

Our findings suggest that TGOC discussions may help reduce infection-related hospitalizations among NH residents, even in facilities with relatively lower levels of engagement in these discussions. Further research is needed to understand how differences in TGOC implementation influence hospitalization decisions. Recognizing the roles of important resident-, facility-, and community-level factors is essential for developing targeted strategies that promote goal-concordant care and reduce avoidable hospital transfers. Ongoing and future efforts to integrate TGOC discussions more systematically into routine NH care while accounting for facility resources and community context may enhance and promote high-quality EOL care for residents and families.

## Authors’ Contributions

All authors contributed to the study concept and design. P.W.S. and A.W.D. contributed to the acquisition of data. All authors contributed to the analysis and interpretation of data, and preparation of article.

## Supplemental Material

sj-docx-1-pmr-10.1177_26892820251410099 — Supplemental material for Association of Timely Goals of Care Discussions with Hospitalizations Due to Infections in Nursing Homes Supplemental material, sj-docx-1-pmr-10.1177_26892820251410099 for Association of Timely Goals of Care Discussions with Hospitalizations Due to Infections in Nursing Homes by
