# Supplementary material for: Association of Timely Goals of Care Discussions with Hospitalizations Due to Infections in Nursing Homes
Source: Palliat Med Rep. 2026 Mar 19;7:26892820251410099. doi: 10.1177/26892820251410099 (PMC13439547; doi:10.1177/26892820251410099)
Supplement: Supplementary file 1 [file sj-docx-1-pmr-10.1177_26892820251410099.docx]

Appendix A

Table 1. Weighted Distribution of TGOC Index Scores (0-18)

| **TGOC category** | **Residents, n (%)** | **Adjusted OR (95 % CI)** | **p value** |
| --- | --- | --- | --- |
| 0-5 (ref) | 96,929 (9.8) | 1.00 | - |
| 6-10 | 136,137 (13.8) | 0.80 (0.66-0.97) | 0.024 |
| 11-15 | 266,711 (27.0) | 0.87 (0.73-1.05) | 0.142 |
| >15 | 488,725 (49.4) | 0.84 (0.71-0.99) | 0.039 |

Abbreviation: TGOC, timely goals of care

Table 2. *Resident, nursing homes, and community characteristics in sample. (867 NHs, 988,502 observations)*

|  |  | **TGOC Index** | | | | |  | | |
| --- | --- | --- | --- | --- | --- | --- | --- | --- | --- |
| Variables | All | 0-5 | 6-18 |  | |  | | | p |
| **TGOC Index (0-18)** Mean (SD) | 13.6 (5.1) | 1.8 (2.0) | 14.8 (3.5) |  | |  | | | *p* < .001 |
| N (%) | 988,502  (100) | 96,929 (9.8) | 891,573  (90.2) |  | |  | | | *p* < .001 |
| **Resident-level Characteristics** | | | | | |  | | |  |
| Race and Ethnicity n (%)* |  |  |  | |  |  | | |  |
| White | 836,152  (84.6) | 79,254  (81.8) | 756,898  (84.9) | |  |  | | |  |
| Black | 83,608  (8.5) | 11,470  (11.8) | 72,138  (8.1) | |  |  | | |  |
| Asian | 22,565  (2.3) | 1,358  (1.4) | 21,207  (2.4) | |  |  | | |  |
| American Indian Alaska Native | 6,700  (0.7) | 191  (0.2) | 6,509  (0.7) | |  |  | | |  |
| Native Hawaiian Pacific Islander | 4,012  (0.4) | 516  (0.5) | 3,496  (0.4) | |  |  | | |  |
| Hispanic | 35,465  (3.6) | 4,140  (4.3) | 31,325  (3.5) | |  |  | | | *p* < .001 |
| Age mean (SD) | 82.2 (8.8) | 81.6 (8.7) | 82.0 (8.8) | |  |  | | | *p* < .001 |
| Female n (%)* | 631,946 | 59,290 | 572,656 | |  | | |  |  |
|  | (63.9) | (61.2) | (63.2) | |  | | |  | *p* < .001 |
| Marital Status n (%)* |  |  |  | |  | | |  |  |
| Never married | 114,910 | 12,248 | 102,662 | |  | | |  |  |
|  | (11.9) | (13.1) | (11.8) | |  | | |  |  |
| Married | 278,289 | 26,635 | 251,654 | |  | | |  |  |
|  | (28.8) | (28.4) | (28.9) | |  | | |  |  |
| Widowed | 445,323 | 41,886 | 403,437 | |  | | |  |  |
|  | (46.1) | (44.6) | (46.2) | |  | | |  |  |
| Separated | 8,998 | 876 | 8,122 | |  | | |  |  |
|  | (0.9) | (0.9) | (0.9) | |  | | |  |  |
| Divorced | 118,766 | 12,210 | 106,556 | |  | | |  |  |
|  | (12.3) | (13.0) | (12.2) | |  | | |  | *p* < .001 |
| CFS n (%)* |  |  |  | |  | | |  |  |
| Intact | 69,055 | 6,749 | 62,306 | |  | | |  |  |
|  | (8.8) | (8.8) | (8.9) | |  | | |  |  |
| Mild impairment | 184,425 | 17,070 | 167,355 | |  | | |  |  |
|  | (23.6) | (22.3) | (23.8) | |  | | |  |  |
| Moderate impairment | 168,585 | 17,037 | 151,548 | |  | | |  |  |
|  | (21.6) | (22.3) | (21.5) | |  | | |  |  |
| Severe impairment | 358,715 | 35,584 | 323,131 | |  | | |  |  |
|  | (45.9) | (46.6) | (45.9) | |  | | |  | *p* < .001 |
| ADL score (0-16) mean (SD) | 6.4 (4.7) | 6.2 (4.7) | 6.4 (4.7) | |  | | |  | *p* < .001 |
| **Facility-level Characteristics** |  |  |  | |  | | |  |  |
| Bed size n (%)* |  |  |  | |  | | |  |  |
| <100 | 309,599 | 32,021 | 277,578 | |  | | |  |  |
|  | (31.3) | (33.0) | (31.1) | |  | | |  |  |
| 100-200 | 526,403 | 53,139 | 473,264 | |  | | |  |  |
|  | (53.3) | (54.8) | (53.1) | |  | | |  |  |
| >200 | 152,500 | 11,769 | 140,731 | |  | | |  |  |
|  | (15.4) | (12.1) | (15.8) | |  | | |  | *p* < .001 |
| Ownership n (%)* |  |  |  | |  | | |  |  |
| For profit | 616,356 | 57,688 | 558,668 | |  | | |  |  |
|  | (62.4) | (59.5) | (62.7) | |  | | |  |  |
| Not-for-profit | 310,517 | 29,259 | 281,258 | |  | | |  |  |
|  | (31.4) | (30.2) | (31.6) | |  | | |  |  |
| Government | 61,629 | 9,982 | 51,647 | |  | | |  |  |
|  | (6.2) | (10.3) | (5.8) | |  | | |  | *p* < .001 |
| Chain affiliated n (%)* | 537,055 | 48,478 | 488,577 | |  | | |  |  |
|  | (54.3) | (50.0) | (54.8) | |  | | |  | *p* < .001 |
| Located in Rural n (%)* | 203,888 | 25,376 | 178,512 | |  | | |  |  |
|  | (20.6) | (26.2) | (20.0) | |  | | |  | *p* < .001 |
| Provider n (%)* |  |  |  | |  | | |  |  |
| Medicare only | 21,430 | 2,491 | 18,939 | |  | | |  |  |
|  | (2.2) | (2.6) | (2.1) | |  | | |  |  |
| Medicaid only | 4,715 | 317 | 4,398 | |  | | |  |  |
|  | (0.5) | (0.3) | (0.5) | |  | | |  |  |
| Medicare and Medicaid | 962,357 | 94,121 | 868,236 | |  | | |  |  |
|  | (97.4) | (97.1) | (97.4) | |  | | |  | *p* < .001 |
| Hours per resident for DONs n (%)* |  |  |  | |  | | |  |  |
| 1^st^ (lower) | 247,174  (25.0) | 21,672  (22.4) | 225,502  (25.3) | |  | | |  |  |
| 2^nd^ | 247,174  (25.0) | 26,889  (27.7) | 220,285  (24.7) | |  | | |  |  |
| 3^rd^ | 247,122  (25.0) | 22,071  (22.8) | 225,051  (25.2) | |  | | |  |  |
| 4^th^ (higher) | 247,032  (25.0) | 26,297  (27.1) | 220,735  (24.8) | |  | | |  | *p* < .001 |
| Hours per resident for RNs n (%)* |  |  |  | |  | | |  |  |
| 1^st^ (lower) | 247,010  (25.0) | 27,553  (28.4) | 219,457  (24.6) | |  | | |  |  |
| 2^nd^ | 247,165  (25.0) | 21,826  (22.5) | 225,339  (25.3) | |  | | |  |  |
| 3^rd^ | 247,163  (25.0) | 19,904  (20.5) | 227,259  (25.5) | |  | | |  |  |
| 4^th^ (higher) | 247,164  (25.0) | 27,646  (28.5) | 219,518  (24.6) | |  | | |  | *p* < .001 |
| Hours per resident for LPNs n (%)* |  |  |  | |  | | |  |  |
| 1^st^ (lower) | 247,023  (25.0) | 25,528  (26.3) | 221,495  (24.8) | |  | | |  |  |
| 2^nd^ | 247,151  (25.0) | 18,901  (19.5) | 228,250  (25.6) | |  | | |  |  |
| 3^rd^ | 247,166  (25.0) | 24,776  (25.6) | 222,390  (24.9) | |  | | |  |  |
| 4^th^ (higher) | 247,162  (25.0) | 27,724  (28.6) | 219,438  (24.6) | |  | | |  | *p* < .001 |
| Resident Occupancy n (%)* |  |  |  | |  | | |  |  |
| 1^st^ (lower) | 247,032  (25.0) | 28,870  (29.8) | 218,162  (24.5) | |  | | |  |  |
| 2^nd^ | 247,156  (25.0) | 19,007  (19.6) | 228,149  (25.6) | |  | | |  |  |
| 3^rd^ | 247,231  (25.0) | 26,319  (27.2) | 220,912  (24.8) | |  | | |  |  |
| 4^th^ (higher) | 247,083  (25.0) | 22,733  (23.5) | 224,350  (25.2) | |  | | |  | *p* < .001 |
| **Community-level Characteristics** | | | | | | | |  |  |
| SDI Score (1-100) mean (SD) | 50.6  (27.0) | 55.9 (25.7) | 50.0 (27.0) |  | | | |  | *p* < .001 |

Note: *column percentage

P values were calculated using Pearson Chi-Square or ANOVA, as appropriate.

Abbreviation: ADL, activities of daily living; CFS, cognitive function scale; SD, standard deviation; SDI, Social Deprivation Index; SES, socioeconomic status; TGOC, timely goals of care;

Table 3. *AOR for hospitalization due to infection in nursing home residents*

|  | | Model | | | |
| --- | --- | --- | --- | --- | --- |
| Variables | | AOR | RSE | 95% CI | |
| **TGOC Index (0-18)** | |  |  |  | |
| 0-5 | | ref |  |  | |
| 6-18 | | 0.84* | 0.07 | 0.72, 0.99 | |
| **Resident-level Characteristics** | |  |  |  | |
| Race and Ethnicity | |  |  |  | |
| White | | ref |  |  | |
| Black | | 1.06 | 0.05 | 0.96, 1.17 | |
| Asian | | 1.02 | 0.10 | 0.84, 1.23 | |
| American Indian Alaska Native | | 1.09 | 0.35 | 0.58, 2.03 | |
| Native Hawaiian Pacific Islander | | 1.10 | 0.25 | 0.70, 1.70 | |
| Hispanic | | 0.97 | 0.09 | 0.80, 1.16 | |
| Age | |  |  |  | |
| 65-75 | | ref |  |  | |
| >75 | | 0.81*** | 0.03 | 0.75, 0.87 | |
| >85 | | 0.65*** | 0.03 | 0.59, 0.71 | |
| Sex | |  |  |  | |
| Male | | ref |  |  | |
| Female | | 0.63*** | 0.02 | 0.59, 0.67 | |
| Marital Status | |  |  |  | |
| Never married | | ref |  |  | |
| Married | | 1.06 | 0.05 | 0.97, 1.16 | |
| Widowed | | 0.97 | 0.05 | 0.88, 1.07 | |
| Separated | | 1.23 | 0.16 | 0.96, 1.58 | |
| Divorced | | 1.01 | 0.06 | 0.91, 1.13 | |
| CFS | |  |  |  | |
| Intact | | ref |  |  | |
| Mild impairment | | 0.06*** | 0.00 | 0.06, 0.07 | |
| Moderate impairment | | 0.15*** | 0.01 | 0.13, 0.17 | |
| Severe impairment | | 0.04*** | 0.00 | 0.04, 0.05 | |
| ADL | |  |  |  | |
| Independent | | ref |  |  | |
| Mild dependence | | 2.04*** | 0.12 | 1.81, 2.29 | |
| Moderate dependence | | 3.56*** | 0.22 | 3.15, 4.02 | |
| Dependent | | 1.38*** | 0.10 | 1.20, 1.60 | |
| **Facility-level Characteristics** | |  |  |  |  |
| Bed size | |  |  |  |  |
| <100 | | ref |  |  | |
| 100-200 | | 1.06 | 0.07 | 0.93, 1.21 | |
| >200 | | 1.10 | 0.14 | 0.86, 1.40 | |
| Ownership | |  |  |  | |
| For profit | | ref |  |  | |
| Not-for-profit | | 0.80*** | 0.05 | 0.71, 0.90 | |
| Government | | 0.66** | 0.09 | 0.51, 0.85 | |
| Chain | |  |  |  | |
| No | | ref |  |  | |
| Yes | | 0.82*** | 0.04 | 0.74, 0.91 | |
| Urban Rural | |  |  |  | |
| Urban | | ref |  |  | |
| Rural | | 0.86* | 0.05 | 0.76, 0.97 | |
| Provider | |  |  |  | |
| Medicare only | | ref |  |  | |
| Medicaid only | | 0.47 | 0.22 | 0.18, 1.17 | |
| Medicare and Medicaid | | 0.94 | 0.15 | 0.69, 1.29 | |
| Hours per resident for DONs (Quartiles) | |  |  |  | |
| 1^st^ (lower) | | ref |  |  | |
| 2^nd^ | | 0.99 | 0.04 | 0.92, 1.07 | |
| 3^rd^ | | 0.99 | 0.04 | 0.92, 1.06 | |
| 4^th^ (higher) | | 0.89 | 0.04 | 0.82, 0.97 | |
| Hours per resident for RNs (Quartiles) | |  |  |  | |
| 1^st^ (lower) | | ref |  |  | |
| 2^nd^ | | 0.97 | 0.04 | 0.90, 1.04 | |
| 3^rd^ | | 0.94 | 0.04 | 0.87, 1.02 | |
| 4^th^ (higher) | | 0.97 | 0.05 | 0.88, 1.07 | |
| Hours per resident for LPNs (Quartiles) | |  |  |  | |
| 1^st^ (lower) | | ref |  |  | |
| 2^nd^ | | 1.03 | 0.04 | 0.95, 1.11 | |
| 3^rd^ | | 1.06 | 0.04 | 0.97, 1.15 | |
| 4^th^ (higher) | | 1.05 | 0.05 | 0.97, 1.15 | |
| Resident Occupancy (Quartiles) | |  |  |  | |
| 1^st^ (lower) | | ref |  |  | |
| 2^nd^ | | 1.03 | 0.04 | 0.94, 1.12 | |
| 3^rd^ | | 0.90 | 0.05 | 0.81, 1.00 | |
| 4^th^ (higher) | | 0.85 | 0.06 | 0.95, 0.97 | |
| **Community-level Characteristics** |  |  |  |  |  |
| Higher SES | | ref |  |  | |
| Moderate-high SES | | 1.08 | 0.08 | 0.93, 1.25 | |
| Moderate SES | | 1.14 | 0.09 | 0.98, 1.32 | |
| Lower SES | | 1.38*** | 0.10 | 1.19, 1.60 | |

Note: **p* < .05; ***p* < .01; ****p* < .001

Abbreviation: ADL, activities of daily living; AOR, adjusted odds ratio; CI, confidence interval; CFS, cognitive function scale; ref, reference; RSE, robust standard errors; SDI, Social Deprivation Index; SES, socioeconomic status; TGOC, timely goals of care; DON, directors of nursing; RN, registered nurses; LPN, licensed practical nurses

Table 4. Sensitivity analysis -*AOR for hospitalization due to infection in nursing home residents*

|  | | Model | | | |
| --- | --- | --- | --- | --- | --- |
| Variables | | AOR | RSE | 95% CI | |
| **TGOC Index (0-18)** | |  |  |  | |
| 0-5 | | ref |  |  | |
| 6-10 | | 0.80* | 0.08 | 0.66, 0.97 | |
| 11-15 | | 0.87 | 0.08 | 0.73, 1.05 | |
| >15 | | 0.84* | 0.07 | 0.71, 0.99 | |
| **Resident-level Characteristics** | |  |  |  | |
| Race and Ethnicity | |  |  |  | |
| White | | ref |  |  | |
| Black | | 1.06 | 0.05 | 0.96, 1.17 | |
| Asian | | 1.02 | 0.10 | 0.84, 1.23 | |
| American Indian Alaska Native | | 1.09 | 0.35 | 0.58, 2.03 | |
| Native Hawaiian Pacific Islander | | 1.10 | 0.25 | 0.70, 1.70 | |
| Hispanic | | 0.97 | 0.09 | 0.80, 1.16 | |
| Age | |  |  |  | |
| 65-75 | | ref |  |  | |
| >75 | | 0.81*** | 0.03 | 0.75, 0.87 | |
| >85 | | 0.65*** | 0.03 | 0.59, 0.71 | |
| Sex | |  |  |  | |
| Male | | ref |  |  | |
| Female | | 0.63*** | 0.02 | 0.59, 0.67 | |
| Marital Status | |  |  |  | |
| Never married | | ref |  |  | |
| Married | | 1.06 | 0.05 | 0.97, 1.16 | |
| Widowed | | 0.97 | 0.05 | 0.88, 1.07 | |
| Separated | | 1.23 | 0.16 | 0.96, 1.58 | |
| Divorced | | 1.01 | 0.06 | 0.91, 1.13 | |
| CFS | |  |  |  | |
| Intact | | ref |  |  | |
| Mild impairment | | 0.06*** | 0.00 | 0.06, 0.07 | |
| Moderate impairment | | 0.15*** | 0.01 | 0.13, 0.17 | |
| Severe impairment | | 0.04*** | 0.00 | 0.04, 0.05 | |
| ADL | |  |  |  | |
| Independent | | ref |  |  | |
| Mild dependence | | 2.04*** | 0.12 | 1.81, 2.29 | |
| Moderate dependence | | 3.56*** | 0.22 | 3.15, 4.02 | |
| Dependent | | 1.38*** | 0.10 | 1.20, 1.60 | |
| **Facility-level Characteristics** | |  |  |  |  |
| Bed size | |  |  |  |  |
| <100 | | ref |  |  | |
| 100-200 | | 1.06 | 0.07 | 0.93, 1.21 | |
| >200 | | 1.10 | 0.14 | 0.86, 1.40 | |
| Ownership | |  |  |  | |
| For profit | | ref |  |  | |
| Not-for-profit | | 0.80*** | 0.05 | 0.71, 0.90 | |
| Government | | 0.66** | 0.09 | 0.51, 0.85 | |
| Chain | |  |  |  | |
| No | | ref |  |  | |
| Yes | | 0.82*** | 0.04 | 0.74, 0.91 | |
| Urban Rural | |  |  |  | |
| Urban | | ref |  |  | |
| Rural | | 0.86* | 0.05 | 0.76, 0.97 | |
| Provider | |  |  |  | |
| Medicare only | | ref |  |  | |
| Medicaid only | | 0.47 | 0.22 | 0.18, 1.17 | |
| Medicare and Medicaid | | 0.94 | 0.15 | 0.69, 1.29 | |
| Hours per resident for DONs (Quartiles) | |  |  |  | |
| 1^st^ (lower) | | ref |  |  | |
| 2^nd^ | | 0.99 | 0.04 | 0.92, 1.07 | |
| 3^rd^ | | 0.99 | 0.04 | 0.92, 1.06 | |
| 4^th^ (higher) | | 0.89 | 0.04 | 0.82, 0.97 | |
| Hours per resident for RNs (Quartiles) | |  |  |  | |
| 1^st^ (lower) | | ref |  |  | |
| 2^nd^ | | 0.97 | 0.04 | 0.90, 1.04 | |
| 3^rd^ | | 0.94 | 0.04 | 0.87, 1.02 | |
| 4^th^ (higher) | | 0.97 | 0.05 | 0.88, 1.07 | |
| Hours per resident for LPNs (Quartiles) | |  |  |  | |
| 1^st^ (lower) | | ref |  |  | |
| 2^nd^ | | 1.03 | 0.04 | 0.95, 1.11 | |
| 3^rd^ | | 1.06 | 0.04 | 0.97, 1.15 | |
| 4^th^ (higher) | | 1.05 | 0.05 | 0.97, 1.15 | |
| Resident Occupancy (Quartiles) | |  |  |  | |
| 1^st^ (lower) | | ref |  |  | |
| 2^nd^ | | 1.03 | 0.04 | 0.94, 1.12 | |
| 3^rd^ | | 0.90 | 0.05 | 0.81, 1.00 | |
| 4^th^ (higher) | | 0.85 | 0.06 | 0.95, 0.97 | |
| **Community-level Characteristics** |  |  |  |  |  |
| Higher SES | | ref |  |  | |
| Moderate-high SES | | 1.08 | 0.08 | 0.93, 1.25 | |
| Moderate SES | | 1.14 | 0.09 | 0.98, 1.32 | |
| Lower SES | | 1.38*** | 0.10 | 1.19, 1.60 | |

Note: **p* < .05; ***p* < .01; ****p* < .001

Abbreviation: ADL, activities of daily living; AOR, adjusted odds ratio; CI, confidence interval; CFS, cognitive function scale; ref, reference; RSE, robust standard errors; SDI, Social Deprivation Index; SES, socioeconomic status; TGOC, timely goals of care; DON, directors of nursing; RN, registered nurses; LPN, licensed practical nurses

Figure 1. Distribution of TGOC Index Scores

Figure 2. Observed infection-related hospitalization rate by TGOC index score
